# Enhanced solution absorption of free-base over protonated nicotine in aerosols

**DOI:** 10.1038/s41598-026-42860-x

**Published:** 2026-03-06

**Authors:** Zhuo Wang, Huapeng Cui, Suxing Tuo, Wen Du, Zhiguo Wang

**Affiliations:** 1 Technology Center, China Tobacco Hunan Industrial Co., Ltd., Changsha, 410072 China; 2 Zhengzhou Tobacco Research Institute of CNTC, Zhengzhou, 450001, China

**Keywords:** Aerosol, Nicotine forms, Absorption pathways, Solution permeation, Chemistry, Drug discovery, Environmental sciences

## Abstract

Elucidating the pharmacokinetic profiles of different chemical forms of nicotine (free-base versus protonated)—particularly their differences in biological absorption—is critically important for balancing its potential clinical applications in neurodegenerative disease therapy with effective management of its inherent addiction risk. Current empirical observations regarding which form exhibits superior absorption remain significantly divergent. This study employs an aerosol delivery model to compare the permeability (simulated absorption) of the two nicotine forms in solution by quantifying the amount absorbed and the residual nicotine after aerosol penetration through a simulated solution barrier. The results demonstrate that free-base nicotine exhibits higher solution absorption than its protonated counterpart. The superior absorption of free-base nicotine is primarily attributed to its diffusion from the particulate phase to the gas phase within aerosols, facilitating efficient mass transfer across the gas–liquid interface. These findings provide an in vitro experimental basis for further evaluation of the potential differences in human absorption efficiency between nicotine forms, thereby supporting the development of nicotine-based pharmaceuticals.

**Supplementary Information:**

The online version contains supplementary material available at 10.1038/s41598-026-42860-x.

## Introduction

Nicotine, as the primary psychoactive component in tobacco, has long attracted widespread attention due to its high addictive potential^1^. The adverse health effects of smoking are primarily attributed to harmful constituents in smoke, such as polycyclic aromatic hydrocarbons, nitrosamines, and tar, which can damage multiple organs including the lungs and heart, and significantly increase the risk of carcinogenesis^2^. In contrast, the core role of nicotine itself lies in its mechanism of addiction: as a potent agonist, nicotine rapidly crosses the blood-brain barrier and acts on nicotinic acetylcholine receptors (nAChRs) in the central nervous system. Due to its relatively slow metabolism, nicotine causes sustained activation of these receptors, leading to receptor desensitization and upregulation, and ultimately resulting in physiological dependence^3^. Beyond physiological dependence, nicotine addiction is also accompanied by prominent withdrawal symptoms, such as anxiety, irritability, poor concentration, and sleep disturbances, which severely disrupt daily life. Additionally, nicotine exerts a direct effect on the cardiovascular system, causing increased heart rate and blood pressure, and elevating the risk of cardiovascular diseases like myocardial ischemia and arrhythmia. Long-term addiction may further pose potential negative impacts on brain cognitive functions (e.g., memory and decision-making abilities), exacerbating health hazards^4^.

However, recent studies have revealed that nicotine’s action on nAChRs may also possess therapeutic potential^5–7^. It can modulate ion channel signaling, promote neurotransmitter release, and activate the cholinergic anti-inflammatory pathway to regulate immune responses, thereby exhibiting neuroprotective properties. Epidemiological^8^ and experimental evidence^9^ suggests that nicotine may have certain efficacy in interventions for neurodegenerative diseases such as Alzheimer’s disease and Parkinson’s disease. It is noteworthy that the biological effects of nicotine exhibit dose dependency: moderate exposure may confer neuroprotection, whereas long-term high-dose exposure tends to induce dependence and neurological damage. Therefore, a thorough understanding of the absorption kinetics of nicotine in the human body is crucial for balancing its therapeutic potential against addiction risks.

The chemical form (protonation state) of nicotine is an important factor influencing its absorption efficiency, yet existing research findings show significant discrepancies. Under physiological conditions, nicotine exists mainly in its unprotonated free base form (I) or mono-/di-protonated salt forms (II/III, i.e., nicotine salts). On one hand, Takano et al.^10,11^ using a rat alveolar epithelial cell model found that increasing extracellular pH enhanced nicotine absorption; Burch et al.^12^ also observed a positive correlation between higher smoke pH and increased nicotine absorption in smokers. These results suggest that the free base form of nicotine has higher bioavailability under alkaline conditions. On the other hand, patent data from Pax Labs^13^ and clinical trials by O’Connell et al.^14^ demonstrated that under the same administration conditions, nicotine benzoate or nicotine lactate salts yielded higher plasma concentrations and faster absorption rates compared to equivalent concentrations of free base nicotine. This implies that salt forms of nicotine may have superior absorption efficacy. The complexity of biological systems has thus far prevented a clear consensus regarding these conflicting results.

To clarify the direct impact of nicotine form on its absorption capacity, this study employed an aerosol delivery model, generating aerosols of free base nicotine and protonated nicotine (nicotine salt), respectively. By measuring the amount of nicotine absorbed and residual after aerosol penetration through a simulated solution barrier, we compared the solution permeability (simulating absorption) of the two nicotine forms, thereby elucidating the fundamental physicochemical processes governing their differential uptake. These findings from the in vitro simulation provide crucial mechanistic evidence to inform and guide subsequent biological investigations, such as those employing epithelial models, toward pharmaceutical applications including the development of neuroprotective agents.

## Experimental

### Experimental materials

Propylene Glycol (99%, China National Pharmaceutical Group Corporation.), Glycerol (99%, China National Pharmaceutical Group Corporation.), Benzoic Acid (99%, China National Pharmaceutical Group Corporation.), Sodium Hydroxide (99%, China National Pharmaceutical Group Corporation.), Hydrochloric Acid (99%, China National Pharmaceutical Group Corporation.), Dichloromethane (99%, China National Pharmaceutical Group Corporation), Deionized Water.

### Experimental methods

Preparation of Test E-liquids: A base vehicle was prepared by mixing propylene glycol and glycerol in a 1:1 mass ratio, followed by the addition of deionized water to achieve a final water content of 5% (w/w). To this base vehicle, the following were separately added:① free-base nicotine, and② nicotine benzoate salt. Both test e-liquids were formulated to a final nicotine mass fraction of 5%.

Preparation of Absorption Solutions: The following absorption solutions (20 mL each) were prepared and placed into individual absorption bottles:① Ethanol;② Ethanol with 1 mmol/L HCl;③ Ethanol with 1 mmol/L NaOH;④ Deionized water (H₂O);⑤ Aqueous solution with 1 mmol/L HCl;⑥ Aqueous solution with 1 mmol/L NaOH.

**Fig. 1:**
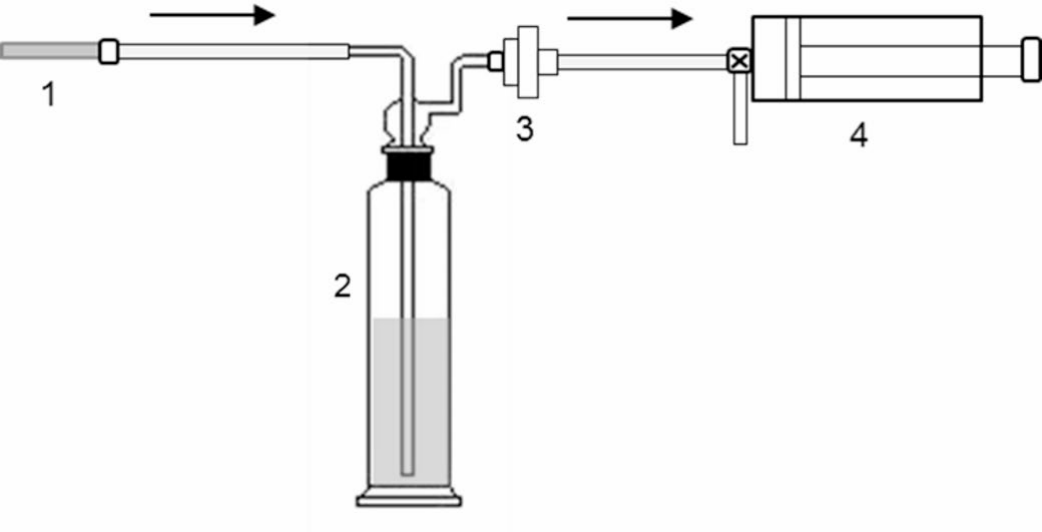
Schematic diagram of the measurement apparatus. 1: Aerosol generator. 2: Absorption bottle. 3: Cambridge filter pad.4: Puffing device.

Experimental Setup and Procedure: The aerosol delivery system was assembled as follows: test e-liquids containing different nicotine forms were loaded into an aerosol generator (The aerosol generator is adapted from the highly mature resistance heating atomization technology used in commercial applications. Its specific working mechanism involves using a controlled electrical current to heat a resistance coil, which rapidly vaporizes the solution in contact with it, thereby generating an aerosol with stable composition. According to prior research, the nicotine concentration in the aerosol under these conditions is a mass fraction of 5%. According to the study by Sosnowski et al.^15^ the particle size distribution of the aerosol in this case conforms to a log-normal pattern, with a mass median diameter of 410 nm.). The generator outlet was connected in series to an absorption bottle (maintained at 37 °C using a water bath) containing 20 mL of absorption solution, a trapping unit equipped with a Cambridge filter pad (Nicotine in the aerosol primarily exists in the particulate phase. The Cambridge filter pad exhibits a retention efficiency exceeding 99.9% for particulate matter with a particle size ≥ 0.3 μm.), and finally a puffing device (Fig. 1).

The puffing device was programmed to operate under a square-wave puff profile with the following parameters: puff volume of 55 mL, puff duration of 3s, and puff interval of 30 s. Each experiment consisted of 20 consecutive puffs.

The procedure was carried out as follows: ① The initial mass of the test e-liquid was recorded: ② The system was connected, and the puffing protocol was initiated.;③ After completion, the system was disconnected, and the test e-liquid was reweighed. The difference in mass was recorded as e-liquid consumption༛④ The Cambridge filter pad was collected and extracted with 10 mL of dichloromethane. The extract was analyzed via gas chromatography–mass spectrometry (GC–MS) to quantify the amount of particulate-phase nicotine trapped༛⑤ For ethanol-based absorption solutions, the solution was directly sampled and analyzed by GC–MS to determine the amount of nicotine absorbed.

To ensure the consistency of instrumental conditions, the solution absorption experiments for nicotine and nicotine salt were conducted simultaneously as a matched set. The entire experimental procedure was subsequently replicated to verify the reproducibility of the results.

## Results and discussion

### Comparison of absorption capacity of different nicotine forms in ethanol-based absorption solutions

**Fig. 2:**
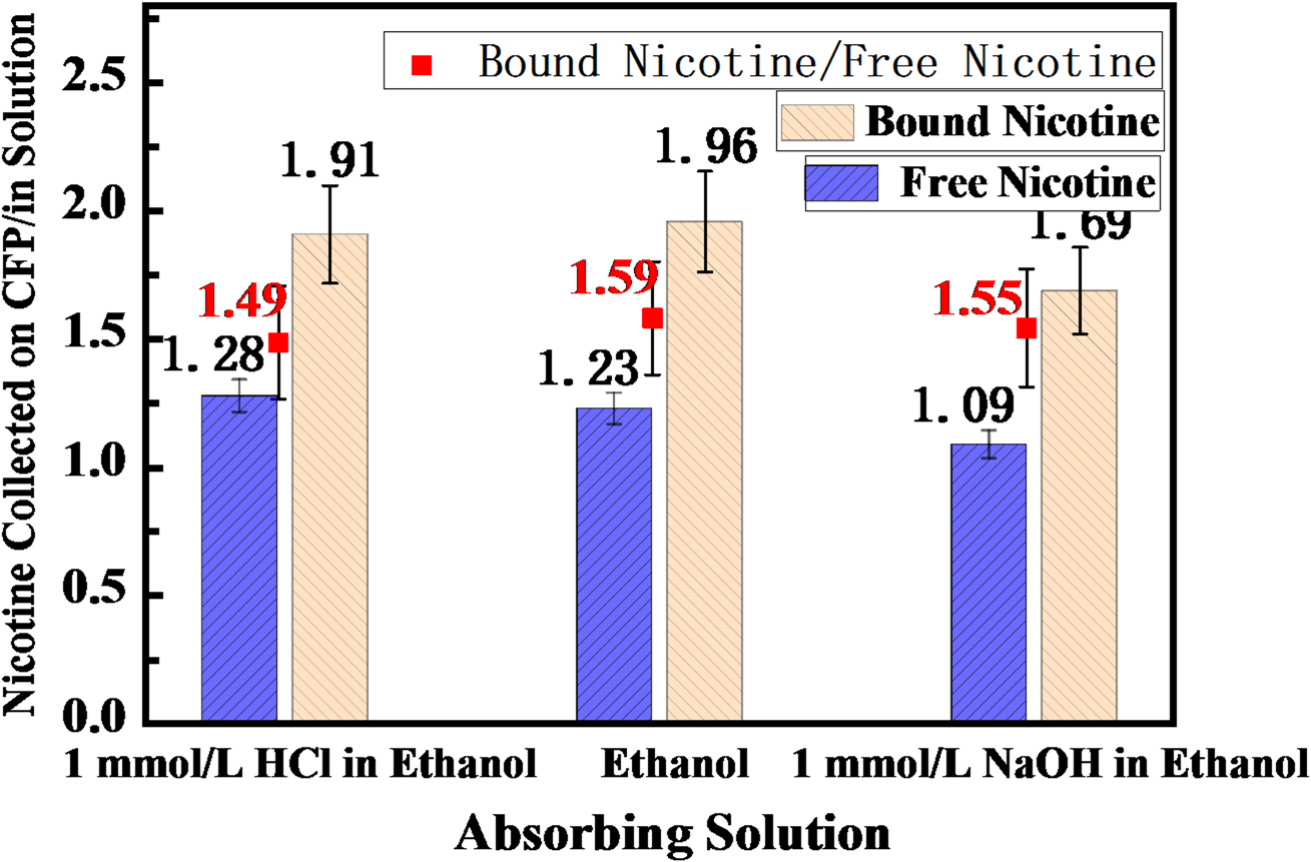
Comparison of absorption of different nicotine forms in ethanol-based absorption solutions across varying pH levels. Blue bars represent the results for free-base nicotine, yellow bars indicate the results for nicotine salt, and red data points illustrate the comparative ratio between the two forms.

Using ethanol as the absorption solution, the captured amounts of different nicotine forms (free-base nicotine and nicotine benzoate) in both the absorption bottle and the Cambridge filter pad were determined. The absorption differences between the two nicotine forms were compared by calculating the ratio of the amount captured on the filter pad to that absorbed in the solution (pad-to-solution nicotine capture ratio). The results are presented in Table 1 and illustrated in Fig. 2. The raw data and data processing procedure are available in the Supporting Information (SI) Table S1.

**Table 1:** Comparison of the absorption of different nicotine forms in ethanolic-based absorption solutions with varying pH values.

Absorption solution	Test E-liquid	Nicotine captured in absorption solution	Nicotine captured on filter pad	Nicotine capture ratio (filter pad/absorption solution)	Mean value ± SD	Nicotine salt/free-base nicotine ratio ± SD
Ethanol	Nicotine 1	1.35	1.71	1.26	1.23 ± 0.04	1.59 ± 0.17
	Nicotine 2	1.52	1.82	1.20		
	Nicotine Salt 1	0.80	1.68	2.10	1.96 ± 0.21	
	Nicotine Salt 2	0.83	1.50	1.81		
1 mmol/L HCl in ethanol solution	Nicotine 1	1.52	1.88	1.24	1.28 ± 0.06	1.49 ± 0.23
	Nicotine 2	1.37	1.81	1.32		
	Nicotine Salt 1	0.72	1.52	2.12	1.91 ± 0.29	
	Nicotine Salt 2	0.89	1.51	1.71		
1 mmol/L NaOH in ethanol solution​	Nicotine 1	1.72	1.90	1.10	1.09 ± 0.02	1.55 ± 0.21
	Nicotine 2	1.80	1.93	1.07		
	Nicotine Salt 1	0.85	1.56	1.85	1.69 ± 0.23	
	Nicotine Salt 2	0.96	1.46	1.52		

The experimental results indicate that under all absorption conditions (ethanol, ethanolic 1 mmol/L HCl solution, and ethanolic 1 mmol/L NaOH solution), the capture efficiency of nicotine salt on the Cambridge filter pad was significantly higher than that of free-base nicotine. This implies that the solution absorption efficiency of free-base nicotine was higher than that of nicotine salt. Specifically, for the ethanolic absorption solution, the ratio of the amount of free-base nicotine captured on the Cambridge filter pad to that absorbed in the solution was 1.23, whereas the ratio for nicotine salt (1.96) was 1.59 times that of the free-base form. This trend was consistently observed in both acidic (ethanolic 1 mmol/L HCl solution) and alkaline (ethanolic 1 mmol/L NaOH solution) absorption media. The corresponding pad-to-solution capture ratios for free-base nicotine were 1.28 (acidic) and 1.09 (alkaline), while those for nicotine salt were 1.91 (acidic) and 1.69 (alkaline). Collectively, these results demonstrate that compared to free-base nicotine, a higher proportion of nicotine salt aerosol particles remained in the aerosol phase (i.e., were captured by the Cambridge filter pad) after passing through the absorption solution, corresponding to a lower absorption efficiency into the solution.

This discrepancy originates from differences in the aerosol kinetic behavior and absorption pathways of the two nicotine forms^16–18^. Free-base nicotine, with lower polarity and higher volatility, readily volatilizes from aerosol particles into the gas phase, subsequently diffusing across the gas-liquid interface and dissolving into the absorption solution—hence its higher solution absorption efficiency. In contrast, nicotine salt, constrained by ionic bonding, exhibits minimal volatility. Its absorption primarily depends on the direct contact and dissolution of nicotine-containing aerosol particles with the absorption solution, resulting in relatively lower solution absorption efficiency.

Furthermore, comparison across absorption solutions with different pH values revealed that the pad-to-solution capture ratios for both free-base and nicotine salt were similar in acidic and neutral solutions (free-base: 1.28 and 1.23; bound-form: 1.91 and 1.96, respectively). However, in the alkaline absorption solution (ethanolic 1 mmol/L NaOH), the ratios decreased for both forms (free-base: 1.09; bound-form: 1.69), indicating enhanced solution absorption of nicotine under alkaline conditions. This phenomenon is related to the polarity of the absorption solution and the distribution of nicotine species. As a weak base, nicotine exists predominantly in the protonated (bound) form under acidic and neutral conditions, whereas the deprotonated (free-base) form becomes predominant under alkaline conditions (1 mmol/L NaOH)^19^. Ethanol, being a weakly polar solvent, exhibits a greater dissolution capacity for molecular organic species (e.g., free-base nicotine) than for ionic compounds (e.g., nicotine salts). Therefore, the increased proportion of the free-base form under alkaline conditions improves the compatibility between nicotine and ethanol, thereby promoting solution absorption.

Notably, however, the ratio of the pad-to-solution capture ratio of nicotine salt to that of free-base nicotine (bound value / free value) did not vary significantly across different pH conditions (acidic: 1.49; neutral: 1.59; alkaline: 1.55). This suggests that the facilitative effect of alkaline conditions on absorption was consistent for both nicotine forms. The bulk absorption solution likely possessed sufficient buffering capacity to maintain the local chemical environment, thereby limiting the influence of the initial chemical form of nicotine on its ultimate dissolution behavior in the solution. This further supports the conclusion that the differences in solution absorption between nicotine forms primarily stem from disparities in aerosol kinetic behavior and absorption pathways, with the intrinsic solubility playing a comparatively minor role.

### Comparison of absorption capacity of different nicotine forms in aqueous-based absorption solutions

**Fig. 3:**
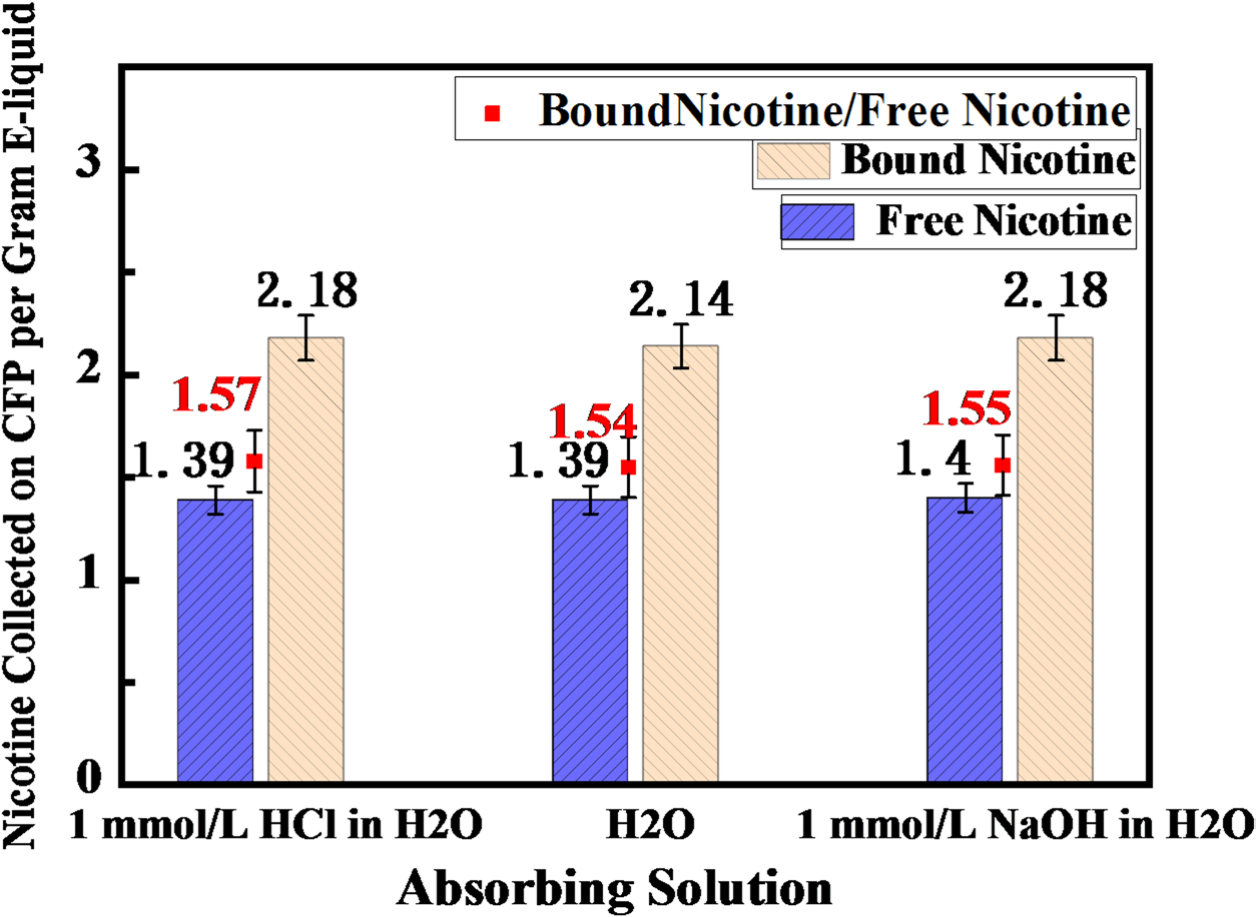
Comparison of absorption of different nicotine forms in H_2_O-based absorption solutions across varying pH levels. Blue bars represent the results for free-base nicotine, yellow bars indicate the results for nicotine salt, and red data points illustrate the comparative ratio between the two forms.

Given that human body fluids are essentially aqueous systems, the aforementioned experiments were repeated using aqueous-based absorption solutions to enhance physiological relevance. Since the measurement methodology employed in this study does not permit direct quantification of nicotine absorbed in aqueous solutions via gas chromatography–mass spectrometry (GC–MS), a normalization approach was adopted to evaluate absorption efficiency. The amount of nicotine captured by the Cambridge filter pad and the total consumption of nicotine solution in each experiment were determined. This allowed for the calculation of the normalized residual amount of nicotine—defined as the quantity of nicotine captured by the filter pad per unit of nicotine solution (or nicotine salt solution) consumed, which represents the fraction of aerosolized nicotine not absorbed by the solution. By systematically comparing this normalized residual amount between different initial forms (free-base nicotine and nicotine salt), the relative solution absorption efficiency in aqueous-based absorption solutions was indirectly assessed (a higher residual value indicates lower solution absorption efficiency). The relevant experimental results are summarized in Table 2 and illustrated in Fig. 3. The raw data and data processing procedure are available in the Supporting Information Table S2.

**Table 2:** Comparison of the absorption capacity of nicotine/nicotine salt in aqueous-based absorption solutions with different pH values.

Absorption Solution	Test E-liquid	E-liquid Consumption (g)	Nicotine Capture on Filter Pad	Nicotine Captured per 1 g E-liquid Consumed	Mean Value ± SD		Nicotine Salt/Free-base Nicotine Ratio ± SD
H_2_O	Nicotine 1	0.128	0.174	1.36	1.39 ± 0.03		1.54 ± 0.04
	Nicotine 2	0.145	0.204	1.41			
	Nicotine Salt 1	0.092	0.197	2.15	2.14 ± 0.02		
	Nicotine Salt 2	0.088	0.188	2.12			
1 mmol/L HCl in H_2_O	Nicotine 1	0.146	0.201	1.38	1.39 ± 0.01		1.57 ± 0.02
	Nicotine 2	0.153	0.214	1.40			
	Nicotine Salt 1	0.090	0.199	2.20	2.18 ± 0.03		
	Nicotine Salt 2	0.093	0.200	2.16			
1 mmol/L NaOH in H_2_O	Nicotine 1	0.146	0.200	1.37	1.40 ± 0.05		1.55 ± 0.06
	Nicotine 2	0.149	0.215	1.44			
	Nicotine Salt 1	0.094	0.204	2.19	2.18 ± 0.01		
	Nicotine Salt 2	0.092	0.201	2.18			

As evidenced by the results presented above, under all aqueous absorption conditions (water, aqueous 1 mmol/L HCl solution, and aqueous 1 mmol/L NaOH solution), the Cambridge filter pad capture efficiency of nicotine salt was significantly higher than that of free-base nicotine. This indicates that the solution absorption efficiency of free-base nicotine is higher than that of nicotine salt. Specifically, when using water as the absorption solution, the ratio of nicotine salt to free-base nicotine captured per unit of nicotine-containing test solution consumed was 1.54, indicating that a greater amount of nicotine salt was retained on the filter pad and thus exhibited lower solution absorption. This trend was consistently observed in both acidic (aqueous 1 mmol/L HCl) and alkaline (aqueous 1 mmol/L NaOH) solutions, with corresponding ratios of 1.57 and 1.55, respectively. In contrast to the observations in ethanolic solutions, the pH of the aqueous absorption solution did not exert a noticeable influence on the relative absorption behavior of the two nicotine forms.

Although the physicochemical properties of the absorption medium (i.e., solvent type and pH) modulated the absolute absorption quantity of nicotine, they did not alter the relative absorption hierarchy between the two chemical forms. This universal behavior stems from the fundamental difference in the mass transfer mechanisms of the two nicotine species. Free-base nicotine, characterized by lower molecular polarity and higher volatility, readily partitions from the aerosol particle phase into the gas phase, subsequently undergoing efficient dissolution into the absorption solution via gas-liquid interfacial diffusion-dominated transfer. Consequently, it demonstrates superior solution absorption performance. In contrast, the absorption of nicotine salt is severely constrained by strong ionic association, which significantly suppresses its volatility. Its release and dissolution are predominantly dependent on direct particle–solution contact and solid–liquid interfacial dissolution kinetics, resulting in systematically lower solution absorption efficiency.

In summary, while environmental parameters such as solvent polarity and pH modulate the absolute extent of nicotine absorption, the chemical state of nicotine (free-base vs. bound-form) remains the central mechanistic determinant of relative absorption efficiency. This is achieved by governing the contribution of two competing mass transfer pathways: volatilization–diffusion versus particle dissolution.

## Conclusion

This study employed an aerosol delivery model to investigate the solution penetration (simulated absorption) capacity of free-base nicotine versus protonated nicotine (nicotine salt). It was demonstrated that free-base nicotine is more readily absorbed into solution compared to its bound-form counterpart. The type of solution (ethanol or water) and its pH value did not alter the relative absorption strength between the two nicotine forms. The observed difference in absorption efficiency is attributed to their distinct absorption pathways: free-base nicotine, due to its ability to transfer from the aerosol particle phase to the gas phase and subsequently diffuse into the solution, achieves higher absorption.

Through the aerosol delivery model, this study confirms that the solution penetration efficiency of free-base nicotine is significantly higher than that of protonated nicotine. The relative absorption strength ratio remained constant across both ethanolic and aqueous systems over a wide pH range, indicating that the physicochemical properties of the absorption medium modulate only the absolute absorption quantity without altering the fundamental inter-form difference. The underlying mechanism originates from the divergence in mass transfer pathways: free-base nicotine, leveraging its lower polarity and higher volatility, undergoes efficient absorption via a multi-stage route of “aerosol particle phase to gas phase to gas-liquid diffusion”. In contrast, protonated nicotine, constrained by ionic bonding, relies solely on a single-rate-limiting pathway of “direct particle-solution contact and dissolution”.

These findings reveal that the protonation state of nicotine governs its interphase transport kinetics by regulating the competition between two pathways—volatilization-diffusion and interfacial dissolution. This study elucidates a key mechanism governing nicotine absorption and provides fundamental in vitro evidence, thereby establishing a critical foundation for future translational research aimed at evaluating its therapeutic potential or addiction risks.

## Supplementary Information

Below is the link to the electronic supplementary material.

Supplementary Material 1

## Data Availability

Data is provided within the manuscript or supplementary information files.
