# Supplementary material for: Enhanced solution absorption of free-base over protonated nicotine in aerosols
Source: Sci Rep. 2026 Mar 6;16:12400. doi: 10.1038/s41598-026-42860-x (PMC13083886; doi:10.1038/s41598-026-42860-x)
Supplement: Supplementary file 1 — Supplementary Material 1 [file 41598_2026_42860_MOESM1_ESM.docx]

Supporting Information

**Enhanced Solution Absorption of Free-Base over Protonated Nicotine in Aerosols**

Zhuo Wang^1^, Huapeng Cui^2^, Suxing Tuo^1^, Wen Du^1^, Zhiguo Wang*^1^

1. Technology Center, China Tobacco Hunan Industrial Co., Ltd., Changsha 410072, China

2. Zhengzhou Tobacco Research Institute of CNTC, Zhengzhou450001, China

*Correspondence to Zhiguo Wang (15656095490@163.com)

**Table of Contents**

**1. Raw data and processing methods of the nicotine experiment based on ethanol absorption solution.**

**2. Raw data and processing methods of the nicotine experiment based on H_2_O absorption solution.**

**1. Raw data and processing methods of the nicotine experiment based on ethanol absorption solution.**

**Table S1. Raw data of the nicotine experiment based on ethanol absorption solution.**

| **Absorption Solution** | **Test E-liquid** | **The Peak Area of the Internal Standard (µV·s)** | | **The Peak Area of Nicotine in the Filter Pad (µV·s)** | **The Peak Area of Nicotine in the Absorption Solution (µV·s)** |
| --- | --- | --- | --- | --- | --- |
| **Ethanol** | **Nicotine 1** | 7.63E+7 | 1.71E+8 | | 6.77 E+7 |
|  | **Nicotine 2** | 7.38 E+7 | 1.82 E+8 | | 7.62 E+7 |
|  | **Nicotine Salt 1** | 7.53 E+7 | 1.68 E+8 | | 3.99 E+7 |
|  | **Nicotine Salt 2** | 7.38 E+7 | 1.50 E+8 | | 4.16 E+7 |
| **1 mmol/L HCl in ethanol solution** | **Nicotine 1** | 7.57 E+7 | 1.88 E+8 | | 7.58 E+7 |
|  | **Nicotine 2** | 7.38 E+7 | 1.81 E+8 | | 6.83 E+7 |
|  | **Nicotine Salt 1** | 7.93 E+7 | 1.52 E+8 | | 3.58 E+7 |
|  | **Nicotine Salt 2** | 7.79 E+7 | 1.51 E+8 | | 4.43 E+7 |
| **1 mmol/L NaOH in ethanol solution​** | **Nicotine 1** | 7.70 E+7 | 1.90 E+8 | | 8.62 E+7 |
|  | **Nicotine 2** | 7.46 E+7 | 1.93 E+8 | | 9.01 E+7 |
|  | **Nicotine Salt 1** | 7.82 E+7 | 1.56 E+8 | | 4.22 E+7 |
|  | **Nicotine Salt 2** | 7.30 E+7 | 1.46 E+8 | | 4.81 E+7 |

Since our study focuses on comparing the absorption intensities of nicotine solutions in different forms, the absolute intensity holds no significance. Here, we directly use the peak areas obtained from the experiments for data processing. The data processing workflow is as follows:

1: Each group of experiments is standardized based on the results of the standard substance to eliminate interference caused by instrument fluctuations during measurement.

2: The relative intensities of nicotine in the Cambridge filter extract and the nicotine absorption solution were calculated based on the amount of sample used in the GC-MS analysis, thereby obtaining the relative intensities of nicotine captured by the filter and absorbed by the solution in each experiment.

3: Based on the relative intensities of nicotine captured by the filter and absorbed by the solution in each experiment, the absorption strength of nicotine in different forms in solutions was calculated.

During data analysis, for the convenience of calculation, we divided all peak area data by 10⁸ before proceeding with further calculations.

**2. Raw data and processing methods of the nicotine experiment based on H_2_O absorption solution.**

**Table S2. Raw data of the nicotine experiment based on H_2_O absorption solution.**

| **Absorption Solution** | **Test E-liquid** | **the Weight of the Aerosol Generator Before the Experiment(g)** | **the Weight of the Aerosol Generator After the Experiment(g)** | | **The Peak Area of the Internal Standard (µV·s)** | **The Peak Area of Nicotine in the Filter Pad (µV·s)** |
| --- | --- | --- | --- | --- | --- | --- |
| **H_2_O** | **Nicotine 1** | 8.173 | 8.045 | 8.49E+07 | | 1.74E+08 |
|  | **Nicotine 2** | 7.454 | 7.309 | 8.34E+07 | | 2.04E+08 |
|  | **Nicotine Salt 1** | 8.096 | 8.004 | 8.28E+07 | | 1.97E+08 |
|  | **Nicotine Salt 2** | 7.637 | 7.549 | 8.11E+07 | | 1.88E+08 |
| **1 mmol/L HCl in H_2_O** | **Nicotine 1** | 8.045 | 7.899 | 8.86E+07 | | 2.01E+08 |
|  | **Nicotine 2** | 7.902 | 7.748 | 8.92E+07 | | 2.14E+08 |
|  | **Nicotine Salt 1** | 8.004 | 7.914 | 8.80E+07 | | 1.99E+08 |
|  | **Nicotine Salt 2** | 7.915 | 7.822 | 8.97E+07 | | 2.00E+08 |
| **1 mmol/L NaOH in H_2_O** | **Nicotine 1** | 7.749 | 7.603 | 8.45E+07 | | 2.00E+08 |
|  | **Nicotine 2** | 7.603 | 7.454 | 8.61E+07 | | 2.15E+08 |
|  | **Nicotine Salt 1** | 7.823 | 7.730 | 8.58E+07 | | 2.04E+08 |
|  | **Nicotine Salt 2** | 7.730 | 7.637 | 8.44E+07 | | 2.01E+08 |

Since our study focuses on comparing the absorption intensities of nicotine solutions in different forms, the absolute intensity holds no significance. Here, we directly use the peak areas obtained from the experiments for data processing. The data processing workflow is as follows:

1: Each group of experiments is standardized based on the results of the standard substance to eliminate interference caused by instrument fluctuations during measurement.

2: The liquid nicotine consumption in each experiment was obtained from the weight difference of the aerosol generator before and after the experiment.

3: The relative intensities of nicotine in the Cambridge filter extract was calculated based on the amount of sample used in the GC-MS analysis, thereby obtaining the relative intensities of nicotine captured by the filter in each experiment.

3: Based on the relative intensity of nicotine captured by the filter and the liquid nicotine consumption in each experiment, the relative intensity of nicotine captured by the filter per unit weight of nicotine was obtained.

4: Compare the solution absorption intensities of nicotine in different forms.

During data analysis, for the convenience of calculation, we divided all peak area data by 10⁸ before proceeding with further calculations.
